# Navigating a vulnerable transition: a qualitative study of the role of companions and providers in pregnancy and childbirth in Burkina Faso

**DOI:** 10.1136/bmjopen-2025-110420

**Published:** 2026-05-07

**Authors:** Emma Wallengren, Kadija Ouedraogo, Claudia Hanson, Helle Mölsted Alvesson, Fadima Yaya Bocoum, Charles Paulin Kaboré, Alexandre Dumont, Ana Pilar Betrán, Amanda Cleeve

**Affiliations:** 1 Department of Global Public Health, Karolinska Institutet, Stockholm, Sweden; 2 Institut de Recherche en Sciences de la Santé, Ouagadougou, Burkina Faso; 3 Department of Disease Control, London School of Hygiene & Tropical Medicine, London, UK; 4 African Population Health Research Center, Dakar, Senegal; 5 CEPED, Institute for Research on Sustainable Development, IRD-Université de Paris, ERL INSERM SAGESUD, Paris, France; 6 UNDP/UNFPA/UNICEF/WHO/World Bank Special Programme of Research, Development and Research Training in Human Reproduction (HRP), Department of Sexual and Reproductive Health and Research, World Health Organization, Geneva, Switzerland; 7 Department of Women's and Children’s Health, Karolinska Institutet, Stockholm, Sweden

**Keywords:** Pregnancy, Patient-Centered Care, Africa South of the Sahara, QUALITATIVE RESEARCH, OBSTETRICS

## Abstract

**Abstract:**

**Objective:**

To explore women’s expectations and experiences of care and support from pregnancy to childbirth in Burkina Faso, with a focus on the role and impact of companions and providers.

**Design:**

An exploratory qualitative study based on in-depth interviews with purposively sampled participants and employing reflexive thematic analysis.

**Setting:**

Two public hospitals in urban Burkina Faso having implemented the ‘QUALIty DECision-making by women and providers for appropriate use of caesarean section’ intervention.

**Participants:**

24 purposively selected postpartum women with variation in terms of parity, mode of birth, labour companionship experiences, education level and occupation were interviewed before discharge from the hospital.

**Results:**

The two themes generated from the analysis elucidate how women rely on providers and companions to navigate uncertainty and vulnerability experienced during pregnancy and childbirth. Women viewed providers as essential for managing the biomedical risks of childbirth and voiced their need for care at critical moments. They expected companions to enhance the non-clinical aspects of their experiences by providing spiritual support and alleviating feelings of loneliness. However, participants also expressed ambivalence about companions witnessing intimate aspects of their birth experience and valued the ability to choose a companion as means to preserve personal integrity.

**Conclusions:**

Both providers and labour companions play an essential role in enhancing women’s experiences of pregnancy and childbirth in Burkina Faso. Additional research and programmatic efforts are needed to support women’s equitable participation in patient–provider interactions and operationalise the notion of choice of a labour companion in a contextually appropriate manner.

**STRENGTHS AND LIMITATIONS OF THIS STUDY**

- The qualitative approach and participants’ diverse experiences with labour companionship enabled an analysis of different aspects of the WHO guidelines, particularly regarding the companion’s continuous presence during labour and birth and women’s choice of companion.
- Interviews were conducted at the hospital shortly after birth. While this may have led to social desirability and limited the depth of some interviews, this also enabled us to sample women from diverse geographic and social contexts.
- Trustworthiness of the analysis was sought by combining insider and outsider perspectives and seeking critical feedback from authors not directly involved in the coding process.

## Background

 Pregnancy and childbirth are meaningful life transitions that can elicit a range of emotions, from hope and joy to uncertainty and vulnerability. Women’s experiences of care and support during this period have important implications for their physical and mental health, as well as future care-seeking behaviours.^1^,^3^ Despite global recognition that women’s experiences of care matter, investments in respectful and woman-centred maternal care are not routinely prioritised, and in some settings, not even considered.^4 5^ Particularly in low-income and middle-income settings, limited resources and political commitment have put interventions to promote women’s experiences of maternity care low on the agenda.^5^ This calls for renewed efforts to understand and prioritise women’s experiences during pregnancy and childbirth.

The experience of childbirth entails both physical and social changes, signifying a meaningful transition in a woman’s life.^6 7^ Globally, rising rates of institutional births have led to safer birth outcomes while exposing women to new vulnerabilities as women’s traditional support systems are transforming.^8 9^ Non-clinical aspects of care, such as effective communication and support during labour, are essential for positive experiences, especially when complications arise.^10^,^12^ Communication with providers not only shapes women’s engagement in care during the perinatal period, but also their approach to future pregnancies.^13^,^15^ Alongside providers’ support, women in various settings also value the presence of a trusted companion during labour and childbirth, which has shown promise to enhance labour progression, birth satisfaction as well as patient–provider interactions.^16 17^

### The QUALI-DEC project

The multifaceted intervention ‘QUALIty DECision-making by women and providers for appropriate use of caesarean section (QUALI-DEC)’ involves four components with the goal of promoting the appropriate use of caesarean sections (C-section): (1) opinion leaders to improve evidence-based clinical practices; (2) audits and feedback to help healthcare providers identify unnecessary C-section; (3) implementation of companionship during labour and childbirth to support parturient women and (4) a decision analysis tool (DAT) to help women make an informed decision on mode of birth.^18^ QUALI-DEC has potential for enhancing women’s pregnancy and birth experiences, particularly through the implementation of the WHO recommendations on labour companionship.^19^ This involves enabling women to choose a companion to provide continuous support throughout labour and childbirth.^19^

The QUALI-DEC research project was implemented in 32 health facilities in four countries: Argentina, Burkina Faso, Thailand and Vietnam. Formative research conducted prior to the implementation of QUALI-DEC indicates that women in Burkina Faso value companionship as an important source of support during labour.^20^ Labour companionship has a long history in Burkina Faso, rooted in a traditional practice of female birth attendants and more recently integrated into a national strategy mandated by the Ministry of Health.^21^ However, persisting barriers limit the practical implementation of companionship, including limited space in the labour and delivery wards, lack of privacy and social norms preventing the choice of companion by women themselves.^20^ Due to these challenges, the current practice of companionship typically deviates from the WHO model in two ways: companions tend to be chosen by the family and their presence is often limited to the first stage of labour, if space allows.^20^

While pregnant and birthing women across contexts express a desire for support, their specific needs and how they should be addressed greatly vary by setting.^22^ In addition, little is known about how women’s needs may change across the perinatal period, particularly during the sensitive transition of childbirth.^23^ In Sub-Saharan Africa, an increasing number of studies have documented poor pregnancy and childbirth experiences,^3 24 25^ but fewer have explored what contributes to positive experiences of care and a sense of support.^5^ In particular, no study to our knowledge has explored women’s lived experiences with the WHO model of labour companionship in Burkina Faso. Understanding how aspects of this model, such as women’s ability to choose their companion and the continuity of the companion’s presence, may influence women’s experiences of support is crucial for providing culturally sensitive care.

The objective of this study is to explore women’s expectations and experiences of care and support from pregnancy to childbirth in Burkina Faso, with a focus on the role and impact of labour companions and providers. Conceptually, this study extends understanding of how women themselves articulate the role of both clinical and non-clinical sources of support during the perinatal period. It also offers practical insight on implementing the WHO guidelines on companionship in a contextually sensitive manner to enhance women’s experiences.

## Methods

### Study design

An exploratory qualitative study using in-depth interviews with purposively sampled postpartum women was conducted. The study is nested within a broader mixed-methods process evaluation of the QUALI-DEC intervention.^18 26^ As part of this process evaluation, qualitative interviews were conducted with key stakeholders towards the end of the implementation phase of the QUALI-DEC project, which lasted approximately 2 years in each participating country. This study draws on data collected in Burkina Faso between November and December 2023. The COnsolidated criteria for REporting Qualitative research (COREQ) checklist was used to guide the reporting of the study.^27^

### Theoretical framework

This study draws on the concept of vulnerability in birth developed by Jennifer MacLellan to understand women’s expectations and experiences of birth.^7^ Drawing on the work of the anthropologist Arnold van Gennep,^6^ MacLellan conceptualises birth as a liminal phase of social and physical transition, opening women to a unique sense of vulnerability. While vulnerability etymologically means exposure to potential harm, MacLellan reframes it as a ‘negative capability‘—the capacity to remain open and present in the face of uncertainty or discomfort, without seeking immediate resolution.^7^ In this view, embracing vulnerability can foster empowerment and relational trust. Applied to childbirth, this perspective suggests that if birthing women, providers and companions allow for vulnerability without immediately seeking to counter it with control or intervention, it can create space for agency, trust and a positive birth experience.^7^ In addition, the framework draws attention to the sociohistorical context shaping women’s experiences of birth vulnerability, including the process of increased medicalisation of birth taking place in both high-income and low-income countries. It provides a theoretical lens to deepen understanding of women’s expectations and experiences as they navigate through pregnancy and hospital-based childbirth in Burkina Faso.

### Study setting

Despite increasingly high rates of institutional birth,^28^ maternal mortality rates in Burkina Faso remain high, with an estimated 242 deaths per 100 000 live births in 2023.^29 30^ In 2020, the percentage of births attended by a skilled birth attendant was estimated at 95.5%.^28^ While the national C-section rate in 2022 was 4%, both underuse and overuse of C-section coexist at the local level, with regional C-section rates 1.6–11.2%.^28 31^ These low C-section rates at the population level may mask suboptimal labour management, including unnecessary primary C-section among women who enter labour spontaneously.^32^

This study was conducted in two purposefully selected public hospitals in urban Burkina Faso. These two hospitals were chosen among eight participating healthcare facilities for an in-depth case study as part of the process evaluation of QUALI-DEC.^26^ Two hospitals, one with a high and one with a medium degree of implementation, were purposively selected using implementation indicators collected during regular monitoring visits to capture complementary stakeholder perspectives on the implementation of QUALI-DEC.^26^ The selected healthcare facilities were a district hospital and a regional hospital, located respectively in Ouagadougou, the country’s capital, and Tenkodogo, the capital city of the Centre-East region of Burkina Faso. Both facilities have a relatively high caseload, a shared labour and delivery ward layout and a limited number of beds for the active phase of labour. The model of care is midwifery-led, with midwives providing care for women throughout labour and childbirth, unless complications arise. Most women giving birth in these hospitals attend antenatal care in lower-level facilities within their local district. C-section deliveries are performed under regional anaesthesia (spinal block).

### Companionship model

The implementation of companionship differs across settings globally.^16^ The QUALI-DEC project aimed to implement the companionship model recommended by the WHO, where a companion chosen by the woman is present continuously throughout labour and childbirth.^19^ This model has been shown to improve maternal and newborn health outcomes as well as women’s satisfaction in different contexts.^33^ In the two selected facilities, companionship was already practised before the implementation of QUALI-DEC.^20^ However, key differences existed compared with the WHO model. First, companionship was offered only to some women, depending on the availability of space in the labour ward.^20^ Additionally, companions were typically present only during labour, and they were often chosen by family members rather than women themselves.^20^ They were not permitted during C-section deliveries due to space constraints.

### Sampling and data collection

In-depth interviews (IDIs) were conducted with postpartum women shortly before they were discharged from the hospital, between one and two days after giving birth. Women who had experienced serious adverse birth outcomes, including stillbirth, neonatal death, severe malformations and severe maternal health problems, were excluded. The sampling approach was guided by Malterud’s concept of information power, which posits that the more relevant information a sample holds, the smaller the required sample size.^34^ Participants were purposively selected to create a diverse sample in terms of mode of birth, parity, labour companionship experiences, education level and occupation. Variation according to these characteristics was considered important to reflect a range of perspectives and generate a nuanced understanding of the study’s aim. The information power of a purposive sample of around 20–24 participants (10–12 per facility), established with specific aspects of variation in mind, was considered adequate to address the objective of the study.

A semi-structured qualitative guide composed of 20 open-ended questions (online supplemental file 1) was developed drawing on the published literature and the study’s objective. The main topics included expectations and preferences regarding childbirth, perceptions and experiences of patient–provider interactions during antenatal care and childbirth, including discussions regarding mode of birth, experiences with labour companionship and how this influenced women’s relationships with providers and sense of support. The interview guide was piloted and refined before data collection. Two female interviewers (FYB, KO) local to the study area and experienced in qualitative research methods conducted the IDIs in French or Mooré, as preferred by the women. The interviewers held a PhD and a master’s degree respectively and were employed as qualitative analysts during the study. They had no prior relationship with participants but initially established rapport by approaching them shortly before the interview and enquiring about their interest in participating. The discussions took place in a private room in the hospital, with only the interviewer and the participant present to ensure confidentiality, and lasted around one hour. The interviewers probed in a flexible manner to further explore topics that emerged from the discussion. The interviews were audio-recorded and simultaneously transcribed and translated into French by a professional third party.

A total of 24 participants, 12 in each hospital, were interviewed. Participants were between 14 and 38 years old, with a mean age of 26. Most had completed secondary education or higher; common occupations included shopkeeper, housekeeper, student and farmer. The majority reported cohabiting with their partner rather than being married. Around two-thirds of the sample were multiparous women. Participants attended between one and eight prenatal consultations during their most recent pregnancy, with most attending at least five. 15 participants gave birth vaginally, six had an intrapartum C-section and three a pre-labour C-section. Of the 16 women who experienced any type of companionship, six had a companion throughout labour and childbirth, and only three stated that they had chosen their companion. Table 1 provides detailed participant characteristics.

**Table 1:** Main characteristics of participants (n=24).

Sociodemographic characteristics	
Age (years)	
Mean	26
Range	14–38
Marital status	
Married	3
Cohabiting	21
Highest level of education	
Primary	8
Secondary	13
Tertiary	1
Unknown	2
Occupation	
Shopkeeper	10
Housekeeper	7
Student	3
Farmer	2
Seamstress	1
Cleaner	1
*Reproductive history*	
Parity	
Primiparous	8
Multiparous	16
History of C-section in previous pregnancies	
Yes	3
No	13
Number of antenatal visits during most recent pregnancy	
1–2	2
3–4	7
5–6	10
7–8	5
Mode of birth for the most recent pregnancy	
Vaginal birth	15
Pre-labour C-section	3
Intrapartum C-section	6
Experiences with labour companionship	
Had a labour companion	16
At some point during labour or childbirth	10
Throughout labour and childbirth	6
Choice of the companion	
Companion chosen by the family	10
Companion chosen by the participant	3
Unclear	3

### Data analysis

A reflexive thematic analysis was conducted following the steps outlined by Braun and Clarke.^35 36^ An experiential orientation to data analysis was adopted, whereby data are conceived as a way into participants’ worlds and experiences.^37^ After an initial reading of the entire dataset to familiarise themselves with the data, two authors (KO, EW) coded the transcripts in French on both semantic and latent levels using the software NVivo. The themes were iteratively developed as outputs of the coding process to capture patterns of shared meaning in the data. The theoretical framing was chosen at this later stage of analysis, helping to shape the underlying narrative that weaves the themes together. Particular attention was given to whether and how two aspects of the WHO recommendations on companionship shape women’s experiences: the choice of companion by women themselves and the continuity of the companion’s presence throughout labour and childbirth.^19^ Among women who did not have a labour companion, the analysis focused on their perceptions of companionship and on how the absence of a companion may have shaped their labour and birth experiences.

Throughout the analysis and manuscript writing process, rigour was sought through regular discussions with co-researchers not directly involved in the coding process (AC, HMA, FYB, CK), who served as ‘critical reviewers’ to encourage alternative interpretations of the data.^38^ The analytical team included researchers from Burkina Faso (FYB, KO, CK) and Sweden (EW, AC, HMA). Integrating both outsider and insider perspectives is a promising strategy to strengthen the reliability of qualitative findings.^39^ While the team prioritised participants’ own interpretations, variation in the lived experiences of the different researchers involved was used to stimulate discussions and broaden perspectives on the data.

### Patient and public involvement

Patient and public involvement was limited to participation in data collection and did not extend to study conceptualisation or analysis. The perspectives of women who had given birth in two public hospitals in Burkina Faso were elicited using qualitative methods, which are well-suited to exploring lived experiences and meaning-making. This approach enabled women to voice their perspectives in their own words to inform improvements in maternal health services in Burkina Faso.

## Results

One overarching theme and two themes were generated. The themes and their corresponding main findings are presented in table 2 and further explored in the narrative below.

**Table 2:** Themes and main findings

Overarching theme: women rely on providers’ and companions’ support to mitigate the uncertainty and vulnerability of childbirth		
Theme 1	Women trust providers to mitigate biomedical risks and voice their need for care in a strained healthcare setting	Women rely on providers to manage the biomedical risks of pregnancy and childbirth Women acknowledge constraints on providers’ time and employ strategies to secure medical attention Companions can facilitate women’s desire for adequate care
Theme 2	Women expect companions to provide emotional and spiritual support but fear compromised personal integrity	The continuous presence of companions helps coping with vulnerability through emotional and spiritual forms of support Companions can increase vulnerability by witnessing and later disclosing intimate aspects of women’s birth experience Women interpret their ability to choose their companion as a safeguard against loss of personal integrity

### Overarching theme

Together, the two themes elucidate how women rely on providers and companions to navigate the uncertainty and vulnerability experienced during pregnancy and childbirth. While they viewed providers as essential for managing the biomedical risks of childbirth, they expected companions to enhance the non-clinical aspects of their experiences through emotional and spiritual support. These expectations became especially salient during the liminal phase of childbirth, when feelings of precariousness and uncertainty were most acute. At these moments, women voiced their need for care and adopted strategies to secure providers’ attention, including relying on companions to communicate their requests. In contrast to the existing model of companionship in Burkina Faso, where companions stay only during the first stage of labour, those who remained throughout childbirth were described as a protective presence, alleviating feelings of loneliness and spiritual vulnerability. However, participants also expressed ambivalence about companions witnessing intimate aspects of their birth experience. In this context, women interpreted their ability to choose a companion as an important safeguard for maintaining personal integrity.

### Theme 1: women trust providers to mitigate biomedical risks and voice their need for care in a strained healthcare context

Women described providers as essential sources of support to mitigate the biomedical risks of pregnancy and childbirth. However, recognising the constraints on providers’ time, they voiced concerns about receiving inadequate clinical attention and care, particularly during the sensitive period of childbirth. To address this, some women employed strategies to secure medical attention and leaned on their companions to facilitate their requests for care.

Women understood childbirth as a biomedically risky event, carrying the prospects of pain, complications and, in the most severe cases, death. Participants’ concerns were often shaped by complications they had experienced during past pregnancies, such as a history of stillbirths or repeated C-sections. Women’s fears also stemmed from their community context, marked by high maternal mortality and intergenerational trauma. Several participants, particularly primiparous women, spontaneously shared stories of women in their family and broader network who had lost their lives in childbirth and described how this fueled their fears about their own pregnancy.

Placing trust in providers was described as essential for managing the health risks of pregnancy and childbirth. Women employed different strategies to gain the support of providers. Continuity of care played an important role, as women valued being followed throughout pregnancy, and ideally, childbirth, by the same provider. Some participants described building long-term relationships with their providers through previous pregnancies or existing connections, such as being neighbours or distant family. Continuity in the patient–provider relationship facilitated trust and enabled women to confide in providers:

She took good care of me. She followed me throughout my pregnancy. (…) She was our neighbor, and since she’s the one who takes care of me, I told her everything. (…) In the evenings when I wasn't working, I called her, and if she was on call I went to see her, and she would explain to me what pregnancies are like. (Participant 2, early 20s, primiparous, pre-labour C-section)

Women valued providers’ medical expertise and guidance, which helped reduce the uncertainties characterising childbirth. Participants described antenatal care as an opportunity to gain information and reassurance on the normal progression of their pregnancy. Some relied on providers’ advice to make concrete behaviour changes that they thought were beneficial to the pregnancy, such as adopting a balanced diet, improving their hygiene and avoiding straining physical tasks. Through the process of seeking and applying providers’ feedback, a few women described gaining increased confidence in their own abilities and bodies. They trusted that diligent engagement in care would help them achieve their ideal of a ‘good birth’—a healthy baby delivered vaginally:

I wasn't afraid because everything was guaranteed. I even had a doctor watching me closely, so I wasn't afraid that it would be a C-section, I had faith that it would be vaginal. (…) I've followed things properly, if the others gave birth on their own, why shouldn't I? (Participant 8, mid-20s, multiparous, intrapartum C-section)

While relying on providers for a safe birth, several participants expressed concerns about receiving inadequate medical attention and care, particularly during the period of childbirth. They contrasted the high patient loads to the limited number of providers, inevitably resulting in some women receiving less attention than others. In this context, participants emphasised the importance of building a trusting relationship with providers to receive care and achieve a safe birth. In the absence of such relationship, some women employed strategies to secure medical attention, such as alerting a specific provider and holding them accountable:

You can see that if you don't know someone here, you're going to suffer. (…) You don't go in here like that, it’s up to me to make friends with the midwives. (…) I saw one who seemed nice and I told her I'm counting on you. (Participant 8, mid-20s, multiparous, intrapartum C-section)

Some participants also described how companions helped them navigate the busy hospital environment and facilitated their interaction with providers. This was particularly salient for the few women who received continuous companionship throughout labour and childbirth. The consistent support of these companions helped bridge the transient and fragmented presence of providers, particularly by relaying women’s requests for care at critical moments:

My companion stayed with me until birth. (…) The (health workers) came and went. When the pain is intense, I tell my stepmother to call the provider and he'll come and see. When he comes, he consults me and says it’s not time yet, then leaves. (Participant 18, late 20s, multiparous, vaginal birth)

### Theme 2: women expect companions to provide emotional and spiritual support but fear a compromised personal integrity

In addition to facilitating women’s requests for care, continuous support from a companion throughout labour and childbirth was important for alleviating emotional and spiritual vulnerability. However, women viewed the proximity of companions as a double-edged sword, potentially amplifying their sense of vulnerability by witnessing and later disclosing intimate aspects of their birth experience. In this context, participants interpreted their ability to choose a companion as essential for safeguarding personal integrity.

Beyond biomedical concerns, women evoked the risks of childbirth as sources of spiritual vulnerability. They described being pregnant and giving birth as being suspended in a precarious state of uncertainty, where they feared that spirits and other evil forces may intervene. In this vulnerable state, many turned to faith and deferred to God to ensure their safety:

To be pregnant is to be between life and death, so you have to put yourself under God’s protection, because no one else can save you. (Participant 2, early 20s, primiparous, pre-labour C-section)

To cope with this sense of vulnerability, women described the continuous presence of a labour companion as an important source of support. While participants who had companions only during the first stages of labour described how they had helped with the logistical aspects of birth, those who had continuous companionship throughout childbirth particularly valued the emotional and spiritual support provided by their companion. Through prayers, encouraging words and comforting touches, companions helped participants feel better equipped to manage their fears and more confident in their body’s ability to give birth:

It helps a lot, because the person will advise you, talk with you so that you leave your worries behind and push on. (Participant 15, early 20s, primiparous, vaginal birth)

The companion tells you not to be afraid, not to put anything else in your head that you're going to give birth by yourself (vaginally). (Participant 13, early 30s, multiparous, vaginal birth)

While women valued the support of their companion, they also expressed ambivalence about having them witness intimate aspects of their birth experience. They interpreted the ability to choose a companion as a safeguard for preserving personal integrity during birth. They voiced their concerns in two main ways. First, participants worried about the companion’s judgement of their behaviours during labour, such as the way they managed pain. This led some to feel self-conscious and regulate how they acted around their companion. In turn, this hindered authenticity and spontaneity, making it difficult for women to stay present and feel like themselves during labour:

Even though I was in too much pain, I was careful about everything I said, as my mother-in-law was there. (Participant 13, early 30s, multiparous, vaginal birth)

I preferred to be alone with the health workers only. It’s better because women can’t be themselves otherwise, others (companions) will come to assist you like that and then it becomes their story, when you make the slightest gesture, they tell you that we saw you when you were giving birth, that’s not what you were doing. (Participant 6, early 20s, multiparous, vaginal birth)

Second, participants expressed concerns that an imposed companion may breach their personal integrity by gossiping about intimate aspects of their experiences after birth. Several women shared stories of how companions, particularly mothers-in-law and co-wives, had wielded birth stories as weapons in arguments. Although most participants did not have the opportunity to choose their companion, all stressed the importance of selecting someone they trusted to feel in control of their birth experience:

During my first delivery, it was a co-wife who accompanied me. So when there’s a disagreement between us, she uses that to say nasty things to me. She says that when I gave birth I was very scared and the health workers couldn’t calm me down. (…) That’s why I say the pregnant woman should choose the person she wants to accompany her. (Participant 13, early 30s, multiparous, vaginal birth)

While most participants were imposed a companion by family members or in-laws, many still expressed satisfaction with their designated companion. Some appreciated the companion’s extensive experience with childbirth, while others evoked a relationship of trust mitigating the risks of loss of privacy:

I didn't want anyone else. If I had the choice, I'd take her because she knows something about childbirth. (Participant 15, early 20s, primiparous, vaginal birth)

Everything was fine (…) since she’s my mom, she’s not going to talk about it, and nobody’s going to hear anything. (Participant 13, early 30s, multiparous, vaginal birth)

## Discussion

This study explored women’s expectations and experiences of support and labour companionship from pregnancy to childbirth in Burkina Faso and in the context of the QUALI-DEC project. Overall, the findings indicate that women rely both on providers and companions to navigate the uncertainty and vulnerability experienced in the perinatal period. Our study extends previous formative research in Burkina Faso^20^ by highlighting how the implementation of the WHO guidelines on companionship may enable companions to facilitate women’s requests for care in a strained hospital environment.

While women valued companionship, the findings also reveal that their expectations of support coexisted with ambivalence and fear of losing personal integrity by having a companion witness this intimate moment. Formative research similarly documented women’s fears that companions may gossip about their birth experience.^20^ Drawing on MacLellan’s framework of vulnerability in birth,^7^ particularly the sub-theme ‘fear of exposure’, our findings indicate that companions may accentuate women’s sense of vulnerability during childbirth. In the absence of trust in their companion, women described a loss of control and authenticity in their birth experience, which led them to regulate their behaviours during labour.

Considering the risk of losing personal integrity, women’s ability to choose a companion emerged as a central yet nuanced finding. While participants expressed a desire to choose their companion, they also discussed the important cultural role of family members in such decisions. This is consistent with population-based estimates indicating that only one in three women in Burkina Faso has primary decision-making power regarding her healthcare.^40^ Yet, in some instances, imposed companions were described as supportive, particularly if they had prior experience of labour companionship and were someone the woman trusted, which raises questions about balancing the risks and benefits of imposed companionship. From an individual rights perspective, more efforts are needed to enhance women’s voice and support their agency in choosing appropriate support. From the angle of cultural appropriateness, future programmes must account for the central role played by family members in selecting a labour companion. These efforts should be sensitive to the powerful gender representations shaping labour companionship practices, acknowledging that these may vary across settings.^41^ A recent meta-analysis including studies from 18 countries globally concluded that the current evidence base is insufficient to provide global recommendations on choosing the ideal companion.^42^ Instead, authors recommend the use of local protocols accounting for cultural beliefs.^42^ The review also found that the level of familiarity between women and their companion was associated with better maternal and neonatal health outcomes, suggesting that beyond choice, relational quality and trust are key to better supporting and meeting women’s expectations.^42^ Our study adds to the growing evidence that eliciting local understandings of what constitutes appropriate support is essential to ensure positive childbirth experiences.

Alongside expectations of support from companions, participants demonstrated awareness of biomedical risks and viewed providers as essential to achieving a safe birth. This finding can be interpreted in the context of increased institutionalisation of maternity care in Burkina Faso and other African settings and the broader literature on the medicalisation of childbirth.^43^,^45^ In a qualitative study by Melberg et al*,* rural Burkinabé women similarly described a generational shift in the perception of childbirth, characterised by heightened concerns about complications and a greater reliance on medical assistance.^9^ To achieve a safe birth, some participants in our study sought to enhance the care they received, including by building a relationship of trust with their provider and voicing their need for care during critical moments. This aligns with other literature on maternal health service utilisation which shows that women are not passive recipients of care but rather actively seek to improve their safety and experiences.^46 47^ An ethnographic study conducted in rural Burkina Faso described women’s use of tactics to enhance their chances of having a positive experience of maternal healthcare and achieve biomedical security.^46^ In our study, however, these exercises of agency were discussed by only a few participants and were restricted to the sensitive period of childbirth. More efforts are needed to enhance the accountability of the health system to enable women to participate more equitably in decision-making throughout the perinatal care continuum.

The findings indicate that implementing the WHO guidelines on companionship may offer a first step toward making the health system more responsive by enabling companions to support women’s requests for care in a resource-limited hospital setting. Through their continuous presence, companions acted as a communication bridge between providers and women and relayed their need for medical attention. The potential of companions to enhance the responsiveness of care has been described in other strained healthcare settings, including in Liberia,^48^ Kenya^49^ and South Africa.^50^ While this presents an opportunity to strengthen companions’ capacity to support positive experiences of maternity care, this role should not be viewed as a substitute for addressing structural issues such as insufficient provider-to-patient ratios or the need for broader investments in the health system.

Finally, this study offers insights into the implementation of QUALI-DEC in Burkina Faso from women’s perspectives, highlighting both challenges and opportunities. Participants responded positively to aspects of the WHO recommendations on labour companionship. They identified additional forms of support enabled by the continuous presence of companions, including spiritual grounding and enhancing their confidence to give birth. However, most women in our study did not have a companion present throughout labour and birth, likely reflecting hospital-level barriers such as staff shortages and time constraints, as well as limited opportunities for women to voice their needs. This aligns with previous studies that have documented how inadequate resources and difficult working conditions impede the delivery of person-centred care in the Burkinabé context.^20 51 52^ In Thailand, barriers to the implementation of the labour companionship component of QUALI-DEC similarly included hospital constraints such as a lack of physical space, overcrowding and restrictive facility policies.^53^ However, some challenges were specific to the Thai context, including providers’ fears of lawsuits and infection, and the belief that companions should not be allowed in the labour room without extensive training.^53^ Together, these findings underscore the need to address local barriers to companionship practices while also strengthening health systems more broadly.

### Limitations and strengths

This study should be interpreted in light of its limitations. First, the short postnatal discharge window constrained us to interview women shortly after giving birth. This may have affected participants’ ability to reflect and limited the richness of the interviews, as some participants showed signs of fatigue. Second, conducting interviews within the hospital may have introduced social desirability and prompted participants to minimise their criticisms of care. Third, some nuances may have been lost in the process of translating parts of the interview transcripts from Mooré to French. However, this risk was mitigated by involving a Mooré-speaking analyst who had also participated in the data collection and who clarified the local meaning of certain expressions.

Nevertheless, this study has several strengths. The diversity of participants’ experiences with companionship enabled an analysis of different aspects of the WHO guidelines, particularly the continuous presence of companions and women’s ability to choose their companion. Including women who did not have a labour companion was also a strength, as it provided a point of comparison to more clearly identify the ways in which the presence of a labour companion may shape labour and birth experiences. The analytical process also benefited from the inclusion of insider and outsider perspectives, which are valuable to achieve both contextual understanding and critical interpretation.^39^ Finally, study rigour was enhanced by soliciting critical feedback from reviewers who were not involved in the initial coding, which helped iteratively refine the analysis and broaden interpretations.^38^

### Implications for policy and practice

To fully realise the benefits of continuous companionship during labour and childbirth in Burkina Faso, additional efforts are required to operationalise the notion of choice in a contextually appropriate manner. National and subnational policies may need to be established or revised to align with WHO recommendations and to create enabling environments within facilities. Simultaneously, community awareness efforts could help promote understanding of the value of companionship of choice, and the importance of respectful behaviour by companions, including maintaining confidentiality and privacy, to reduce the social or privacy-related concerns associated with imposed companions. Such interventions would support women’s emotional well-being and autonomy while respecting the local sociocultural context.

## Supplementary material

10.1136/bmjopen-2025-110420 online supplemental file 1

10.1136/bmjopen-2025-110420 online supplemental file 2

## Data Availability

Data are available upon reasonable request.
