# Supplementary material for: Navigating a vulnerable transition: a qualitative study of the role of companions and providers in pregnancy and childbirth in Burkina Faso
Source: BMJ Open. 2026 May 7;16(5):e110420. doi: 10.1136/bmjopen-2025-110420 (PMC13157755; doi:10.1136/bmjopen-2025-110420)
Supplement: online supplemental file 1 [file bmjopen-16-5-s001.docx]

INDIVIDUAL INTERVIEWS: POSTPARTUM WOMEN

STUDY AIM AND OBJECTIVES

The overall aim of this study is to explore patient-provider relationships (including dynamics and communication) and perceptions on quality of care during pregnancy, labour, and childbirth, among postpartum women.

ELIGIBILITY

| **ELIGIBILITY CRITERIA** | **YES** | **NO** |
| --- | --- | --- |
| Experience with labour companionship during labour and/or birth: |  |  |
| Experience with the DAT |  |  |
| Did the woman have a stillbirth? |  |  |
| Was the baby born alive but dies after (neonatal death)?  *If the woman had a multiple pregnancy, did any of the babies die?* |  |  |
| Did the woman have a baby with malformation? *If the woman had a multiple pregnancy, did any of the babies have malformations?* |  |  |
| Did the woman present major health problems?  *(i.e., severe postpartum haemorrhage, severe cardiac illness, mental disease, near miss and any life-threatening conditions based on medical records/consultation with local clinician)* |  |  |

**INTERVIEW GUIDE**

MODULE 1: SOCIO-DEMOGRAPHIC BACKGROUND AND REPRODUCTIVE HISTORY (filled by interviewer)

| Age |  |
| --- | --- |
| Education | [ ] Incomplete primary school  [ ] Complete primary school  [ ] Incomplete secondary school  [ ] Complete secondary school  [ ] Some or completed tertiary education |
| Occupation |  |
| Marital status | [ ] Married  [ ] Cohabiting  [ ] Single  [ ] Divorced  [ ] Widowed |
| Total number of births |  |
| Total vaginal births |  |
| Total CS |  |
| Number of antenatal visits for most recent pregnancy |  |
| Mode of birth for most recent pregnancy | [ ] Vaginal unassisted  [ ] Vaginal assisted (forceps, vacuum)  [ ] CS after labour start  [ ] CS before labour start |

*Interviewer to read: Thank you for agreeing to participate in this interview. Just to remind you, there are no right or wrong answers to these questions; we are interested in your personal views and experiences. I would like to hear from you about your pregnancy and birth. If we start with when you first came to know that you were pregnant…*

MODULE 1: EXPECTATIONS AND PREFERENCES

1. During your pregnancy, how did your thoughts go about giving birth ­ - what were your expectations? Hopes? Fears?

Probes:

- Something you were looking forward to or feared?
- Did they change over time during pregnancy? If so, how, and why?
- Discussed these with your family/partner/friends?

1. What were your thoughts about having a vaginal birth or CS? Any preference for one or the other?
   - Did it change over time? If yes, how, and why?
   - Previous experience (personal/relatives/friends)?
   - Perceived risks/benefits of CS/Vaginal birth

MODULE 2: ANC: COMMUNICATION/PATIENT-PROVIDER RELATIONSHIP

1. Can you tell me about the care received during pregnancy? Did you visit an antenatal care clinic?

Probes:

- Who did you see there?
- How often did you go?

1. When you went to your antenatal care appointments, did you talk about what you wanted for this birth with your physician or midwife?

If yes, can you tell me about how the/se conversation/s went?

Probes:

- Did you talk about your expectations? Needs? fears or doubts?
- What questions did you ask?
- What did the provider tell you?
- Did you have any unanswered questions after the conversation/s?
- How did it feel to talk about these things with your physician/midwife?
  - Could you voice your opinions/ talk about expectations, needs, fears, or doubts?

If NO, probe for reasons why and then go to Q6

1. During your antenatal care visits, did you ever talk about mode of birth?

If yes, can you tell me about how the/se conversation/s went?

Probes:

- What questions did you ask?
- What did the provider tell you?
- Did you have any unanswered questions after the conversation/s?
- How did it feel to talk about these things with your physician/midwife?
  - Could you voice your opinions/ talk about expectations, needs, fears, or doubts?
- Were there any conflicts with the provider? Was it resolved? How?

If no, can you tell me what your expectations were for this birth?

The DAT (keep a copy of the DAT booklet to show the respondent)

1. During your pregnancy did you hear about a tool that informs about the risks and benefits of vaginal birth and caesarean section?

*If no – skip to Q11 MODULE 3*

Probes:

- Who told you about it? When?
- Timing of when the DAT was introduced – was this a good time? Would you have wanted earlier/later?
- What did you hear about it?

1. Did you read the information in the tool (booklet or phone application)?

If yes, what did you think about it?

Probes:

- What parts did you read?
- What did you like about it?
  - Format
  - Content
- Any parts were not so helpful?
- Did you learn anything new from it?

*If no – skip to Q9*

1. What were your thoughts and expectations for this birth after reading the booklet/information in the app?

Probes:

- Did it influence in any way your expectations, fears, or doubts you may have had about giving birth? Thoughts on:
  - Mode of birth, assisted and unassisted vaginal birth, CS.
  - Pain management
  - Birthing positions
  - Type of support you needed/wanted and from whom and when.
  - Labour companionship
  - Breast feeding

1. What was it like discussing and planning for your birth together with your healthcare provider after reading the information in the booklet/app?

Probes:

- What questions did you ask?
- What did the provider tell you?
- Did you have any unanswered questions after the conversation/s?
- How did it feel to talk about these things with your physician/midwife?
  - Could voice your opinions/talk about your needs and expectations, fears, or doubts?
- If you discussed mode of birth - was the provider open to discuss different options?

1. Do you have any other comments or feedback about using the tool?

MODULE 3: LABOUR AND BIRTH

*Ok so let’s move on to talk a bit about your experience of labour and birth in this health facility.*

1. Can you tell me about what happened when you arrived at this facility up until now that we are having this conversation?

Labour companionship

- 1. Some women have a person with them during labour and when giving birth, and we call this person a “labour companion”. Did anyone ask you if you wanted to have someone with you during labour/birth?

12.2 If no, would you have liked a labour companion? Why? Why not?

*Go to Q17*

12.3 If yes, can you tell me about the moment when you first learnt about the option of labour companionship?

Probes:

- From whom?
- When during pregnancy?
- What were you told about labour companionship?
  - The role of the companion
  - Conditions or circumstances for when you could/couldn’t have a companion.
  - Who could be a companion and when
  - Preparations for companions

*If given the option but didn’t end up having a labour companion at any stage:*

- 1. What were the reason/s for why you didn’t end up having a labour companion?

*Go to Q18*

*If she had a labour companion at any stage:*

1. Can you tell me how and when was it decided who should accompany you?

Probes:

- Who chose?
- Easy decision? Difficult? Why?
- Did you discuss this decision with anyone?

1. Where was your companion during your labour? When the baby came out? Do you recall what was he/she doing?

Probes:

- Was he/she next to you? Why or why not?
- What was he/she doing?
- Was he/she there all the time or for part of your labour or birth? How come?

1. Where was the nurse/midwife/physician during your labour? During birth? Do you recall what was he/she doing?

Probes:

- Was he/she next to you? Why or why not?
- What was he/she doing?
- Was he/she there all the time or for part of your labour or birth?

1. What was it like having a labour companion during labour and/or birth?

Probes:

- Can you give some examples of what you liked about having a companion? What didn’t you like?
- Would you recommend it to your friends? Why? Why not?

1. What could have been done to improve your experience with having a labour companion?

Probes:

- Choice of companion
- Being able to choose when/for how long the companion could stay.
- Facilities for labour and birth - privacy
- How the provider spoke to the companion
  - Practical instructions for the companion
  - Keeping the companion informed
  - Being able to ask questions.

Birth experience - general

1. Was there anything about your labour and birth and your time here in this healthcare facility that you didn’t like so much? Can you give some examples?

Probes:

- Time/Prescence of healthcare provider
- Type of support/timing of support
- Information about the labour and birth process
- Information about interventions* – why certain things were needed/done/not done/recommended.
- Asked to consent prior to interventions*?
- Instructions and communication
- Privacy

**With interventions we mean things that were done during labour and birth such as administering a certain pill or injection, rupturing membranes, checking the position of the baby, examining the cervical dilatation, listening to the baby's heartbeat, changing positions, vacuum extraction and so forth.*

1. Was there anything about your labour and birth and your time in this healthcare facility that you liked especially? Can you give some examples?

Probes - See Q18

1. Is there anything that you would like to add?

*Thank you for your time!*
