# Supplementary material for: Navigating a vulnerable transition: a qualitative study of the role of companions and providers in pregnancy and childbirth in Burkina Faso
Source: BMJ Open. 2026 May 7;16(5):e110420. doi: 10.1136/bmjopen-2025-110420 (PMC13157755; doi:10.1136/bmjopen-2025-110420)
Supplement: online supplemental file 2 [file bmjopen-16-5-s002.docx]

ENTRETIENS INDIVIDUELS: FEMMES EN SUITES DE COUCHES

BUT ET OBJECTIFS DE L’ETUDE

L'objectif général de la présente étude est d'analyser les relations patiente-prestataire de soins (y compris la dynamique et la communication) et les perceptions de la qualité des soins pendant la grossesse, le travail et l'accouchement, chez les femmes en suites de couches.

*L'enquêteur/trice lit : Merci d'avoir accepté de participer à cet entretien. Je vous rappelle qu'il n'y a pas de bonnes ou de mauvaises réponses à ces questions. Ce qui nous intéresse, ce sont vos opinions et vos expériences personnelles. J'aimerais que vous me parliez de votre grossesse et de votre accouchement. Commençons par le moment où vous avez appris que vous étiez enceinte.*

MODULE 1: ATTENTES ET PREFERENCES

1. Pendant votre grossesse, comment avez-vous envisagé l'accouchement, quelles étaient vos attentes? Vos espoirs? Vos craintes?

Questions pour approfondir:

- Quelque chose que vous attendiez avec impatience ou que vous redoutiez ?
- Ces attentes, espoirs et craintes ont-ils évolué au fil de la grossesse? Si oui, comment et pourquoi ?
- En avez-vous discuté avec votre famille/partenaire/vos amis ?

1. Quels étaient vos sentiments par rapport à un accouchement par voie basse ou par césarienne? Aviez-vous une préférence pour l'un ou l'autre?
   - Cette préférence a-t-elle évolué au fil du temps ? Si oui, comment et pourquoi ?
   - Expérience antérieure (personnelle/parents/amis) ?
   - Risques/avantages perçus de la césarienne/de l'accouchement par voie basse.

MODULE 2: CPN: COMMUNICATION/RELATION PATIENTE-PRESTATAIRE

1. Pouvez-vous me parler des soins reçus pendant la grossesse ? Vous êtes-vous rendue dans un centre de soins prénatals ?

Questions pour approfondir:

- Qui avez-vous vu là-bas ?
- Combien de fois y êtes-vous allée ?

1. Lorsque vous êtes allée à vos rendez-vous de soins prénatals, avez-vous parlé de ce que vous vouliez pour cet accouchement avec votre médecin ou votre sage-femme ?

Si oui, pouvez-vous me dire comment s'est/se sont déroulée(s) la/les conversation(s) ?

Questions pour approfondir:

- Avez-vous parlé de vos attentes? De vos besoins? De vos craintes ou de vos doutes?
- Quelles questions avez-vous posées?
- Que vous a dit le/la prestataire de soins?
- Avez-vous eu des questions sans réponse après la/les conversation(s) ?
- Qu'avez-vous ressenti en parlant de ces choses avec votre médecin/sage-femme?
  - Avez-vous pu exprimer vos opinions/parler de vos attentes, de vos besoins, de vos craintes ou de vos doutes ?

Si NON, cherchez à savoir pourquoi et passez à Q6.

1. Au cours de vos consultations prénatales, avez-vous déjà parlé du mode d'accouchement ?

Si oui, pouvez-vous me dire comment s'est / se sont déroulée(s) cette/ces conversation(s) ?

Questions pour approfondir:

- Quelles questions avez-vous posées?
- Que vous a dit le/la prestataire de soins?
- Avez-vous eu des questions sans réponse après la/les conversation(s)?
- Qu'avez-vous ressenti en parlant de ces choses avec votre médecin/sage-femme?
  - Avez-vous pu exprimer vos opinions/parler de vos attentes, de vos besoins, de vos craintes ou de vos doutes ?
- Y a-t-il eu des conflits avec le/la prestataire de soins? Ont-ils été résolus? Comment ont-ils été résolus ?

Si non, pouvez-vous me dire quelles étaient vos attentes pour cet accouchement?

Le DAT (conservez un exemplaire de la brochure du DAT pour le présenter à la répondante)

1. Pendant votre grossesse, avez-vous entendu parler d'un outil d'information sur les risques et les avantages de l'accouchement par voie basse et par césarienne ?

*Si non, passez à Q11 MODULE 3*

Questions pour approfondir:

- Qui vous en a parlé? Quand?
- La période d'introduction du DAT était-elle propice? Auriez-vous voulu que ce soit plus tôt ou plus tard?
- Qu'avez-vous entendu à ce sujet?

1. Avez-vous lu les informations contenues dans l'outil (brochure ou application téléphonique)? Si oui, qu'en avez-vous pensé?

Questions pour approfondir:

- Quelles parties avez-vous lues ?
- Qu'est-ce qui vous a plu?
  - Le format
  - Le contenu
- Y a-t-il des parties qui n'ont pas été très utiles?
- Avez-vous appris quelque chose de nouveau?

*Si non, passez à Q9*

1. Quelles étaient vos sentiments et vos attentes à l'égard de cet accouchement après avoir lu la brochure/les informations contenues dans l'application ?

Questions pour approfondir:

- Cela a-t-il influencé d'une manière ou d'une autre vos attentes, vos craintes ou vos doutes concernant l'accouchement ? Vos sentiments sur :
  - Le mode d'accouchement, l'accouchement par voie basse avec ou sans assistance, la césarienne.
  - La gestion de la douleur
  - Les positions d'accouchement
  - Le type de soutien dont vous aviez besoin ou que vous souhaitiez, de qui et quand.
  - Le compagnonnage pendant le travail
  - L'allaitement maternel.

1. Comment s'est déroulée la discussion et la planification de votre accouchement avec votre prestataire de soins après avoir lu les informations contenues dans la brochure/l'application ?

Questions pour approfondir:

- Quelles questions avez-vous posées ?
- Que vous a dit le/la prestataire de soins?
- Avez-vous eu des questions sans réponse après la/les conversation(s)?
- Qu'avez-vous ressenti en parlant de ces choses avec votre médecin/sage-femme?
  - Avez-vous pu exprimer vos opinions/parler de vos besoins et de vos attentes, de vos craintes ou de vos doutes ?
- Si vous avez abordé le mode d'accouchement, le/la prestataire de soins était-il/elle ouvert(e) à l'idée de discuter de différentes options?

1. Avez-vous d'autres remarques ou réactions concernant l'utilisation de l'outil?

MODULE 3: TRAVAIL ET ACCOUCHEMENT

*Bien, parlons donc un peu de votre expérience du travail et de l'accouchement dans cette formation sanitaire.*

1. Pouvez-vous me raconter ce qui s'est passé depuis le moment où vous êtes arrivée dans cette formation sanitaire jusqu'en ce moment où nous tenons cette conversation?

Compagnonnage pendant le travail

- 1. Certaines femmes sont accompagnées d'une personne pendant le travail et l'accouchement, et nous appelons cette personne un/une « compagnon/ne de travail ». Vous a-t-on demandé si vous souhaitiez avoir quelqu'un avec vous pendant le travail/l'accouchement?
  2. Si non, auriez-vous aimé avoir un/une compagnon(ne) de travail? Pourquoi? Pourquoi pas?

*Allez à Q17*

- 1. Si oui, pouvez-vous me parler du moment où vous avez découvert l'option du compagnonnage?

Questions pour approfondir:

- De la part de qui?
- A quel moment au cours de la grossesse ?
- Que vous a-t-on dit sur le compagnonnage pendant le travail?
  - Le rôle du/de la compagnon(ne)
  - Les conditions ou les circonstances dans lesquelles vous pouvez ou ne pouvez pas avoir un/une compagnon(ne).
  - Qui pourrait être un/une compagnon(ne) et quand?
  - Les préparatifs pour les compagnons(nes).

*Si l'option vous a été présentée mais que vous n'avez pas eu recours à un/une compagnon(ne) de travail à un moment ou à un autre :*

- 1. Pour quelle(s) raison(s) n'avez-vous pas eu recours à un/une compagnon(ne) pendant le travail?

*Allez à Q18*

*Si elle a eu un/une compagnon(ne) de travail à un moment ou à un autre :*

1. Pouvez-vous me dire comment et quand il a été décidé qui devait vous accompagner ?

Questions pour approfondir:

- Qui a choisi?
- Décision facile à prendre ? Difficile? Pourquoi?
- Avez-vous parlé de cette décision avec quelqu'un d'autre?

1. Où était votre compagnon(ne) pendant le travail? Lorsque le bébé est sorti ? Vous souvenez-vous de ce qu'il/elle faisait?

Questions pour approfondir:

- Etait-il/elle à côté de vous? Pourquoi ou pourquoi pas?
- Que faisait-il/elle ?
- Etait-il/elle présent(e) tout le temps ou pendant une partie du travail ou de l'accouchement? Pourquoi?

1. Où était l'infirmier (ère)/la sage-femme/le médecin pendant le travail? Pendant l'accouchement? Vous souvenez-vous de ce qu'il/elle faisait?

Questions pour approfondir:

- Etait-il/elle à côté de vous ? Pourquoi ou pourquoi pas?
- Que faisait-il/elle ?
- Était-il/elle présent(e) tout le temps ou pendant une partie du travail ou de l'accouchement?

1. Qu'est-ce que cela vous a apporté d'avoir un/une compagnon(ne) de travail pendant le travail et/ou l'accouchement?

Questions pour approfondir:

- Pouvez-vous donner des exemples de ce que vous avez aimé dans le fait d'avoir un/une compagnon(ne)? Qu'est-ce qui ne vous a pas plu?
- Recommanderiez-vous cela à vos amies ? Pourquoi? Pourquoi pas?

1. Qu'est-ce qui aurait pu être fait pour améliorer votre expérience de compagnonnage pendant le travail?

Questions pour approfondir:

- Le choix du/de la compagnon(ne)
- La possibilité de choisir le moment et la durée de la présence du/de la compagnon(ne).
- Les infrastructures destinées au travail et à l'accouchement, l'intimité
- La façon dont le/la prestataire de soins s'est adressé(e) au/à la compagnon(ne)
  - Instructions pratiques à l'intention du/de la compagnon(ne)
  - Informations données au/à la compagnon(ne)
  - Possibilité de poser des questions.

Expérience de l’accouchement - généralités

1. Y a-t-il des aspects de votre travail, de votre accouchement et de votre séjour dans cette formation sanitaire que vous n'avez pas appréciés? Pouvez-vous donner quelques exemples?

Questions pour approfondir:

- Temps/présence du/de le prestataire de soins
- Type de soutien / opportunité du soutien
- Informations sur le déroulement du travail et de l'accouchement
- Informations sur les interventions, pourquoi certaines choses étaient nécessaires/ont été faites/non faites/recommandées.
- Consentement requis avant les interventions ?
- Instructions et communication
- Intimité.

**Nous entendons par interventions ce qui a été fait pendant le travail et l'accouchement*

1. Y a-t-il des aspects de votre travail et de votre accouchement et de votre séjour dans cette formation sanitaire que vous avez particulièrement appréciés? Pouvez-vous donner quelques exemples?

Questions pour approfondir. Voir Q18

1. Y a-t-il quelque chose que vous aimeriez ajouter?

*Merci de m'avoir accordé votre temps!*
