# Supplementary material for: Navigating a vulnerable transition: a qualitative study of the role of companions and providers in pregnancy and childbirth in Burkina Faso
Source: BMJ Open. 2026 May 7;16(5):e110420. doi: 10.1136/bmjopen-2025-110420 (PMC13157755; doi:10.1136/bmjopen-2025-110420)
Supplement: Supplementary file 3 [file bmjopen-2025-110420.reviewer_comments.pdf]

## PEER REVIEW HISTORY

BMJ Open publishes all reviews undertaken for accepted manuscripts. Reviewers are asked to complete a checklist review form and are provided with free text boxes to elaborate on their assessment. These free text comments are reproduced below.

### ARTICLE DETAILS

**Title (Provisional)**

Navigating a vulnerable transition: a qualitative study of the role of companions and providers in pregnancy and childbirth in Burkina Faso

**Authors**

Wallengren, Emma; Ouedraogo, Kadija; Hanson, Claudia; Alvesson, Helle Molsted; Bocoum, Fadima Yaya; Kabore, Charles; Dumont, Alexandre; Betran, Ana Pilar; Cleeve, Amanda

---

### VERSION 1 - REVIEW

---

| | |
|---|---|
| **Reviewer** | **1** |
| **Name** | **Malapela, Rakgadi** |
| **Affiliation** | **University of South Africa** |
| **Date** | **14-Oct-2025** |
| **COI** | **None** |

---

A major revision is required to improve the quality of the study. Comments have been attached for your revision and resubmission

### Technical presentation

Avoid writing in 1st person, like using "we", and write in 3rd person

Lines 1-3: The title is too long; consider shortening it and adhering to the author guidelines.

Keywords:

Identify the key words in line with the research title, such as life and death, pregnancy, labour companionship, hospital childbirth, etc.

## ABSTRACT:

Purpose: Rephrase the purpose

Line 32-34: To explore women's expectations and experiences of care and support from pregnancy to childbirth in Burkina Faso, with a focus on the role and impact of having a companion and provider during childbirth

Line 35:

### Design

Line 146: Specify the research design method.

### Results

Line 44-45: The two themes generated from the analysis elucidate how women rely on providers and companions to navigate the uncertainty and vulnerability experienced during pregnancy and childbirth. How about adding the role and impact of providers to the purpose so that the results align with it?

## BACKGROUND:

Line 88-89: Suggest that the background starts with an open statement without a source.

Line 105: Add the purpose and contribution to emphasise the significance of this study.

140-143: Maintain consistency in stating the aim of this study. Follow author Guudleines by using correct wording, whether it is the objectives or aim of the study

## METHODS

Line 146: Specify the Research design used. Line 203:

Under-sampling and data collection

In accordance with Domain 2 of the COREQ Checklist, most of the information was omitted. Duration of the interview, when data saturation was reached. Who was present during the interview besides the participants? Were there any dropouts from the participants? The pretesting of the interview guide was not mentioned.

Line 237: Table Characteristics of participants

To be included under the results section and not under the methods section 238: Data

analysis

Line 249-250: Specify the data analysis steps followed, as guided by whom?

## RESULTS:

Line 263: Results

Start with the demographic characteristics of your participants to improve the flow before introducing the themes and sub-themes

Suggest that you add the participant number, e.g. “P1”, in the following Lines: Line 308,

321, 332, 340, 356, 366, 368, 379, 383, 394, 401 & 403

## ETHICS APPROVAL

Line 545: Specify whether consent was obtained verbally, in writing, or both.

## REFERENCES

Line 550. Maintain consistency in the referencing technique, taking into consideration the author guidelines. Line 571-572, page numbers were included in brackets. Other references, pages were not included in Line 591, 601, 605, 609 & 614.

---

| | |
|---|---|
| **Reviewer** | **2** |
| **Name** | **Kelly, Janet** |
| **Affiliation** | **University of Adelaide, School of Nursing** |
| **Date** | **18-Dec-2025** |
| **COI** | **None** |

---

Thank you for the opportunity to read this paper on a very important topic. The paper is informative and contributes to the existing body of work in supporting women's childbirth and implementing WHO guidelines.

Abstract reads well

Background – provides clear rationale for the project

The theoretical framework brings a new perspective to this space.

The study setting is explained well for an international audience.

Recruitment, selection, interview process, interview and analysis processes are clearly outlined.

I have a few suggestions for minor changes.

Given that half of the results focuses on health care providers and the other half on companions, and both are in the discussion, I suggest slight change to the heading to reflect this. This might be:

"To be pregnant is to be between life and death": a qualitative study of women's reliance on providers and companions for support during pregnancy and hospital based childbirth in Burkina Faso

Or similar.

Results

The two themes are well explained with quotes. These also provide insights into local culture and contexts.

I am however a little confused by the differing participant descriptions used for quotes. Could these be standardised and explained.(What is Pissy- a personal name, a pseudonym, a place? )

(21 years old, primiparous, pre-labor C- section)

(29 years old, multiparous, vaginal birth, IDI 6 Tenkodogo)

(Pissy 1, 21 years old, primiparous, pre-labor C-section)

Re publication 42 ( line 434)– can you indicate which countries are included in this meta analysis. If global, a slight change in the sentence can indicate this.

On line 444- 445 – this can also be interpreted in the wider context of other African countries that are similarly experiencing medicatilization of childbirth and are exploring disrespect and abuse occurring in childbirth facilities. See below for example – there are many others.

Adinew, Y. M., Kelly, J., Smith, M., & Marshall, A. (2023). Women's perspectives on disrespect and abuse during facility-based childbirth in Ethiopia: a qualitative study. BMC Pregnancy and Childbirth, 23(1), 444. doi:10.1186/s12884-023-05762-8.

Discussion

Given the focus on caesarean sections in the wider study, was there any specific discussion or quotes relating companions present during caesarean sections. Also are C/S performed under general anesthetic, spinal block or other in these settings? Is there room and for additional people (companion) in spaces where C/S are performed?

References, relevant, recent.

---

## VERSION 1 - AUTHOR RESPONSE

| Reviewer | Reviewer's comment | Authors' response | Line # (clean manuscript) |
|---|---|---|---|
| Reviewer 1 | Technical presentation<br>Avoid writing in 1st person, like using "we", and write in 3rd person | Thank you – the sentences using first-person were revised to third-person. | l. 127-128, 140-142, 144-145, 156-157, 169-170, 182-183, 194-197, 199-200, 203-207, 236, 242, 258-259, 406, 408, 413, 461, 471, 489, 498, 505-507 |
| Reviewer 1 | Lines 1-3: The title is too long; consider shortening it and adhering to the author guidelines. | We have shortened the title. We were unable to find specific guidelines for title length in this journal. | l. 1-2 |
| Reviewer 1 | Keywords: | Thank you for your comment. BMJ Open uses MeSH terms to | NA |

| | | | |
|---|---|---|---|
| | Identify the key words in line with the research title, such as life and death, pregnancy, labour companionship, hospital childbirth, etc. | index articles. We included Pregnancy and Labor, Obstetric but were unable to identify MeSH terms specifically related to labour companionship or hospital-based childbirth. Life and death are emic terms used by women in our study and do not have corresponding MeSH terms. | |
| Reviewer 1 | ABSTRACT:<br>Purpose: Rephrase the purpose<br>Line 32-34: To explore women's expectations and experiences of care and support from pregnancy to childbirth in Burkina Faso, with a focus on the role and impact of having a companion and provider during childbirth | We have integrated the suggested change by mentioning both companions' and providers' role in the objective. We made this change both in the abstract and in the background. | l. 37-38, 127-128 |
| Reviewer 1 | Design<br>Line 146: Specify the research design method. | As suggested, we have now specified that the study employs an exploratory qualitative design, and clarified its data collection method (in-depth interviews) and analytical approach (reflexive thematic analysis). We have made this change in the abstract and in the Methods section. | l. 39-40, 135-136 |
| Reviewer 1 | Results<br>Line 44-45: The two themes generated from the analysis elucidate how women rely on Providers and companions to navigate the uncertainty and vulnerability experienced during pregnancy and childbirth. How about adding the role and impact of providers to the purpose so that the results align with it? | As suggested, we have now rephrased the objective to mention both companions' and providers' role. We made this change both in the abstract and in the Background section. | l. 37-38, 127-128 |
| Reviewer 1 | BACKGROUND:<br>Line 88-89: Suggest that the background starts with an open statement without a source. | As suggested, we have added an open sentence introducing women's experiences of pregnancy and childbirth as meaningful transitions encompassing a range of emotions. | l. 72-73 |
| Reviewer 1 | Line 105: Add the purpose and contribution to emphasize the significance of this study. | We have now added two sentences describing the conceptual and programmatic contributions of the study. These were added after the objective rather than earlier in the background, as we felt it was | l. 128-132 |

| | | | |
|---|---|---|---|
| | | helpful to first introduce the QUALI-DEC project and the WHO guidelines on companionship to contextualize the study's contributions. | |
| Reviewer 1 | 140-143: Maintain consistency in stating the aim of this study. Follow author Guidelines by using correct wording, whether it is the objectives or aim of the study | We have aligned the phrasing of the study's objective to ensure consistency between the abstract and background. We used the term "objective" consistently in accordance with the BMJ Open submission guidelines. | l. 36-38, 126-128 |
| Reviewer 1 | METHODS:<br>Line 146: Specify the Research design used. | We have now described the research design as an exploratory qualitative study and further specified the data collection method (in-depth interviews). In the Data analysis section, we specified the analytical approach (reflexive thematic analysis). | l. 135-136, 234-235 |
| Reviewer 1 | Line 203: Under-sampling and data collection<br>In accordance with Domain 2 of the COREQ Checklist, most of the information was omitted. Duration of the interview, when data saturation was reached. Who was present during the interview besides the participants? Were there any dropouts from the participants? The pretesting of the interview guide was not mentioned. | We used the concept of information power (Malterud, 2015), rather than data saturation, to guide our choice of sample size. Considering the focused nature of our research objective and the specific sample of women with relevant experiences of both provider support and labor companionship, we estimated that a sample of 20-24 participants was adequate.<br><br>We have now added information on the pre-testing of the guide.<br><br>We specified that only the interviewer and the participant were present during the interview and that the discussion lasted approximately one hour.<br><br>We did not track the number of women who were approached to participate in the interview. In line with our qualitative research approach, the sampling strategy was not intended to minimize selection bias or achieve representativeness, but rather to identify participants able to provide rich accounts of their experiences. | Sample size: l. 197-205<br><br>Pre-testing: l. 211-212<br><br>Duration of IDIs and who was present: l. 217-219 |

| | | | |
|---|---|---|---|
| Reviewer 1 | Line 237: Table Characteristics of participants<br>To be included under the results section and not under the methods section | Thank you for your comment. We suggest retaining the paragraph and table describing participant characteristic in the Methods section. This is consistent with qualitative research conventions in the social sciences, where participant characteristics are considered part of the context that shapes meaning-making. Presenting this information in the Methods section signals that they frame the study rather than constitute findings and allows readers to assess the study context and quality before reading the results. | NA |
| Reviewer 1 | 238: Data analysis<br>Line 249-250: Specify the data analysis steps followed, as guided by whom? | We specified that we conducted a reflexive thematic analysis as conceptualized by Braun and Clarke. We revised parts of the Data analysis section to more clearly describe the steps of our analytical process in line with this approach. | l. 235-242 |
| Reviewer 1 | RESULTS:<br>Line 263: Results<br>Start with the demographic characteristics of your participants to improve the flow before introducing the themes and sub-themes | Thank you for this comment. As explained in our earlier response, we suggest retaining this information in the Methods section to provide context that frames the results rather than constitutes findings, consistent with qualitative research conventions in the social sciences. | NA |
| Reviewer 1 | Suggest that you add the participant number, e.g. "P1"<br>, in the following Lines:<br>Line 308, 321, 332, 340, 356, 366, 368, 379, 383, 394, 401 & 403 | We have added participant numbers as suggested. | l. 308, 321-322, 332, 341, 357, 367, 369, 380, 384, 395, 402, 404 |
| Reviewer 1 | ETHICS APPROVAL:<br>Line 545: Specify whether consent was obtained verbally, in writing, or both. | We specified that written informed consent was obtained for all participants. | l. 546-548 |
| Reviewer 1 | REFERENCES:<br>Line 550. Maintain consistency in the referencing technique, taking into consideration<br>the author guidelines. Line 571-572, page numbers were included in brackets. Other | We have reviewed the references and ensured they followed AMA style (11$^{th}$ edition). | l. 552-717 |

| | | | |
|---|---|---|---|
| | references, pages were not included in Line 591, 601, 605, 609 & 614. | | |
| Reviewer 2 | ABSTRACT:<br>Abstract reads well<br>Background – provides clear rationale for the project<br>The theoretical framework brings a new perspective to this space.<br>The study setting is explained well for an international audience.<br>Recruitment, selection, interview process, interview and analysis processes are clearly outlined.<br>I have a few suggestions for minor changes. Given that half of the results focuses on health care providers and the other half on companions, and both are in the discussion, I suggest slight change to the heading to reflect this. This might be:<br>"To be pregnant is to be between life and death": a qualitative study of women's reliance on providers and companions for support during pregnancy and hospital based childbirth in Burkina Faso<br>Or similar. | Thank you for your comments.<br><br>We have integrated your suggestion on the title and mentioned providers' role to better align with the results. | l. 1-2 |
| Reviewer 2 | RESULTS:<br>The two themes are well explained with quotes. These also provide insights into local culture and contexts.<br>I am however a little confused by the differing participant descriptions used for quotes. Could these be standardised and explained.(What is Pissy- a personal name, a pseudonym, a place? )<br>(21 years old, primiparous, pre-labor C- section)<br>(29 years old, multiparous, vaginal birth, IDI 6 Tenkodogo)<br>(Pissy 1, 21 years old, primiparous, pre-labor C-section) | Thank you for your comment on the findings.<br><br>We agree with the suggested change regarding the participant descriptions for the quotes. Pissy and Tenkodogo are the names of the hospitals – we deleted these for enhanced confidentiality and clarity. We added participant numbers to distinguish the quotes without identifying the women. | l. 308-309, 321-322, 332-333, 341-342, 357-358, 367, 369-370, 380, 384-385, 395-396, 402, 404 |
| Reviewer 2 | DISCUSSION:<br>Re publication 42 ( line 434)– can you indicate which countries are included in this meta | We specified that this was a global review including studies from 18 countries. | l. 434 |

| | | | |
|---|---|---|---|
| | analysis. If global, a slight change in the sentence can indicate this. | | |
| Reviewer 2 | On line 444- 445 – this can also be interpreted in the wider context of other African countries that are similarly experiencing medicalization of childbirth and are exploring disrespect and abuse occurring in childbirth facilities. See below for example – there are many others. Adinew, Y. M., Kelly, J., Smith, M., & Marshall, A. (2023). Women's perspectives on disrespect and abuse during facility-based childbirth in Ethiopia: a qualitative study. BMC Pregnancy and Childbirth, 23(1), 444. doi:10.1186/s12884-023-05762-8. | We modified the sentence to note that this phenomenon is occurring across other African settings. We added the suggested reference, as well as another study on women's experiences of medicalization and obstetric violence in Ghana. | l. 446-447 |
| Reviewer 2 | Given the focus on caesarean sections in the wider study, was there any specific discussion or quotes relating companions present during caesarean sections. Also are C/S performed under general anesthetic, spinal block or other in these settings? Is there room and for additional people (companion) in spaces where C/S are performed? | The participants did not discuss this as companions are not allowed to be present during CS delivery in Burkina Faso. We specified in the Methods section that CS are performed under spinal block and that labor companions are not permitted. | l. 179-180<br>l. 191-192 |
| Reviewer 2 | REFERENCES:<br>References, relevant, recent. | Thank you. | NA |
